# The Role of Peroxisome Proliferator-Activated Receptor γ in Immune Responses to Enteroaggregative *Escherichia coli* Infection

**DOI:** 10.1371/journal.pone.0057812

**Published:** 2013-02-28

**Authors:** Casandra W. Philipson, Josep Bassaganya-Riera, Monica Viladomiu, Mireia Pedragosa, Richard L. Guerrant, James K. Roche, Raquel Hontecillas

**Affiliations:** 1 Nutritional Immunology and Molecular Medicine Laboratory, Virginia Bioinformatics Institute, Virginia Tech, Blacksburg, Virginia, United States of America; 2 Center for Modeling Immunity to Enteric Pathogens, Virginia Tech, Blacksburg, Virginia, United States of America; 3 Department of Biomedical Sciences and Pathobiology, Virginia-Maryland Regional College of Veterinary Medicine, Virginia Tech, Blacksburg, Virginia, United States of America; 4 Division of Infectious Diseases and International Health, Center for Global Health, University of Virginia, Charlottesville, Virginia, United States of America; Università degli Studi di Milano, Italy

## Abstract

**Background:**

Enteroaggregative *Escherichia coli* (EAEC) is recognized as an emerging cause of persistent diarrhea and enteric disease worldwide. Mucosal immunity towards EAEC infections is incompletely understood due in part to the lack of appropriate animal models. This study presents a new mouse model and investigates the role of peroxisome proliferator-activated receptor gamma (PPARγ) in the modulation of host responses to EAEC in nourished and malnourished mice.

**Methods/Principal Findings:**

Wild-type and T cell-specific PPARγ null C57BL/6 mice were fed protein-deficient diets at weaning and challenged with 5×10^9^cfu EAEC strain JM221 to measure colonic gene expression and immune responses to EAEC. Antigen-specific responses to *E. coli* antigens were measured in nourished and malnourished mice following infection and demonstrated the immunosuppressive effects of malnutrition at the cellular level. At the molecular level, both pharmacological blockade and deletion of PPARγ in T cells resulted in upregulation of TGF-β, IL-6, IL-17 and anti-microbial peptides, enhanced Th17 responses, fewer colonic lesions, faster clearance of EAEC, and improved recovery. The beneficial effects of PPARγ blockade on weight loss and EAEC clearance were abrogated by neutralizing IL-17 *in vivo*.

**Conclusions:**

Our studies provide *in vivo* evidence supporting the beneficial role of mucosal innate and effector T cell responses on EAEC burden and suggest pharmacological blockade of PPARγ as a novel therapeutic intervention for EAEC infection.

## Introduction

Enteroaggregative *E. coli* (EAEC) is a Gram-negative, rod-shaped bacterial pathogen of the *Enterobacteriaceae* family recognized as an emerging causative agent of gastroenteritis and diarrhea in developing and industrialized countries worldwide [1], [2]. EAEC infections can cause diarrheagenic symptoms in immunocompromised adults, travelers, victims of food borne illness [3], and particularly severe cases in children with malnutrition [4], [5]. The relationship between malnutrition and diarrheagenic infection has been described as a vicious cyclic pattern hindering the host’s ability to clear bacteria and ameliorate disease [6]. Malnutrition predisposes individuals to infection by impairing epithelial barrier integrity and suppressing immune responses [7]. Adverse effects to intestinal absorption are exacerbated during infection generating a catabolic state that depletes nutrients needed for tissue synthesis and growth further increasing the likelihood of pathogens breaching the epithelial barrier [8]. Malnutrition impairs host responses thereby amplifying infection and pathology [9]. More importantly, EAEC infections hinder the functionality of the epithelial barrier disrupting nutrient absorption worsening malnutrition and potentiating growth retardation [10].

*E. coli* pathovars use a multi-step scheme for pathogenesis consisting of mucosal colonization, evasion of host defenses, replication, and host damage. Direct contact with the epithelium is a key determinant of the host’s innate immune response to EAEC [11]. Specifically, AAF fimbriae are presumably the primary pathognomonic virulence factor contributing to the manifestation of EAEC infection. Aggregated adherence to enterocytes by means of the AAF fimbriae fosters an environment prone to increased colonization. Upon aggregating, EAEC has the capability to disrupt epithelial tight junctions, subsequently leading to penetration of bacterial toxins and induction of the host’s mucosal immune response [12]. Interaction between EAEC flagellin and Toll-like receptor 5 on host epithelial cells elicits a proinflammatory response extensively characterized by secretion of IL-8 from epithelial cells [13], [14]. Proinflammatory responses induced by EAEC are thought to contribute to the pathogenesis of EAEC. IL-8, a principal chemoattractant for polymorphonuclear leukocytes and the migration of these cells into the intestinal mucosa, is a hallmark of inflammatory infectious diarrhea including EAEC-induced disease [15]. Recruitment and transmigration of neutrophils to the gut mucosa causes intestinal damage that may promote EAEC colonization [16]. The role of T cells, dendritic cells (DC) and macrophages in mucosal responses to EAEC remains incompletely understood.

The mucosal immune system in the intestine peacefully coexists with 100 trillion commensal bacteria while responding swiftly to pathogens such as EAEC. These studies aimed to characterize the role of mucosal inflammatory and effector responses during acute EAEC infection and their relation to clinical recovery in a mouse model of malnutrition-induced immunosuppression. We targeted the transcription factor peroxisome proliferator activated receptor (PPAR) γ pharmacologically and genetically to modulate mucosal inflammation and immunity [17] during EAEC infection to evaluate initiation, progression and outcomes. Specifically, we used the compound 2-chloro-5-nitrobenzanilide (GW9662), a potent PPARγ antagonist [18], and conditional PPARγ knockout mice to delineate the impact of PPARγ during infection with EAEC in nourished and malnourished mice.

## Methods

### Animal Procedures

Wild-type, PPARγ tissue-specific conditional knockout mice exhibiting Cre recombinase targeted to the CD4 promoter (PPARγ fl/fl, CD4-cre+) or hematopoietic and epithelial cells (PPARγ fl/fl MMTV-cre+) in a C57BL/6 background were weaned at 21 days of age and assigned to groups that were fed regular purified AIN-93G rodent diet (20% protein) or protein deficient diet (2% protein) (Table S1). Three days post weaning each mouse was challenged intragastrically by gavage with 5×10^9^ CFU EAEC strain JM221. In follow up studies C57BL/6 mice were administered GW9662 (0.5, 1, or 2 µM dose; 13.8 mg/kg, 27.6 mg/kg, and 55.3 mg/kg respectively); Cayman Chemical, Ann Arbor, MI) orogastrically beginning at the time of infection and continuing daily for up to seven days post infection. Anti-IL17A neutralizing antibody (50 µg; R&D Systems, Minneapolis, MN) was administered intraperitoneally on days 0, 2, and 4. Body weights and disease activities were monitored daily. Fecal collection for bacterial shedding quantification was performed.

### Ethics Statement

All experimental procedures were approved by the Virginia Tech Institutional Animal Care and Use Committee (IACUC) (Protocol Number: 10–087VBI) and met or exceeded requirements of the Public Health Service/National Institutes of Health and the Animal Welfare Act. Animals were under strict monitoring throughout the duration of infection and all efforts were made to minimize suffering. Mice were euthanized by carbon dioxide narcosis followed by secondary cervical dislocation.

### Histopathology

Colonic sections were fixed in 10% buffered neutral formalin, later embedded in paraffin, sectioned (6 µm) and stained with H&E. Tissue slides were examined in an Olympus microscope (Olympus America Inc., Dulles, VA). Colons were scored for leukocyte infiltration, epithelial erosion, and mucosal thickness.

### RNA Isolation and Real-time Polymerase Chain Reaction of Cytokines

Total RNA from colon was isolated using the Qiagen RNA isolation kit (Qiagen) according to manufacturer's instructions, and used to generate the cDNA template using iScript cDNA synthesis kit (Bio-Rad, Hercules, CA). Real-time RT-PCR was performed as previously described [19]. Oligonucleotide sequences for the primers used are presented in Table S2.

### DNA Isolation and Quantification of EAEC from Feces

Fecal samples were weighed and DNA was isolated using the QIAamp DNA Stool Mini Kit (Qiagen) according to manufacturer’s instructions. Primer sequences were designed using the Beacon Designer (PREMIER Biosoft) and used to quantify the dispersin gene (*aap)*,using real-time RT-PCR. The *aap* primer sequences for EAEC quantification are presented in Table S2. The protocol for standard cycling ran as follows: 1) 95°C, 5 min 2) 95°C, 30 sec 3) 61.5°C, 30 sec 4) 72°C, 40 sec (repeat step 2 for 40 cycles) 5) Repeat steps 2–4 39 more times 6) 72°C, 10 min 7) Melt curve 65–95°C increment 0.5°C for 5 seconds, plate read. The correlation between DNA quantification data and CFU values was developed as follows: DNA was isolated from eight EAEC JM221 cultures with concentrations of 10 CFU up to 10^8^, each increasing by a factor of 10. Real-time RT-PCR was ran to quantify *aap* in DNA from the known CFU cultures. An eight point standard curve was generated that converted DNA quantities to CFU values. CFU values were divided by the weight of the feces (in milligrams) to obtain a final value for CFU/mg feces.

### Bacterial Growth

EAEC strain JM221 was streaked onto LB Agar (Fisher) and left to grow for 24 hours at 37°C in a static incubator. One colony was then picked from the plate and used to inoculate 5 mL LB media (Fisher) with 0.5% dextrose to create a preinoculum which grew in a shaking incubator at 37°C for 12 hours. The preinoculum was used to inoculate a larger volume of LB media (1∶1000 dilution). Optical density (OD) measurements were monitored over time at 600 nm. When bacteria reached optimal growth, media was centrifuged at 4000 rpm for 10 minutes and bacteria pellets were resuspended in LB media at a concentration of 2×10^10^ cfu/mL. Mice received 0.1 ml of the inoculum.

### Flow Cytometry

Colonic lamina propria mononuclear cells and whole blood were seeded onto 96-well plates and used for flow cytometry staining. Cells were incubated in the dark at 4°C for 20 min with the following fluorochrome-conjugated primary antibodies: CD4-PECy7, CD3 PeCy5, IL-10 PE, IL-17A-PerCPCy5.5, IFNγ-PE, MHC-II Biotin-Texas Red, CD11b AlexaFluora700, F4/80 PeCy5, Gr1 PeCy7, CD11c FITC. Flow results were computed using a BD LSR II flow cytometer and data analysis was performed with FACS Diva software (BD).

### Lymphocyte Proliferation Assay

Splenocytes were stimulated in 96-well round bottom plates with media alone (non-stimulated) or medium containing enteroaggregative *Escherichia coli* (EAEC) strain JM221 sonicated antigens, *Escherichia coli* strain HS (non-pathogenic) and mutant Enteroaggregative *Escherichia coli* strain JM221 *Aff^−^* (lacking Aff1 fimbria) whole cell antigens (5 µg/mL). Concanavalin A (5 µg/mL) was used as a positive control for prolferation. Antigen-specific proliferation was measured on day 5 of culture. Cultures were pulsed for the last 20 h with 0.5 µCi of [^3^H]-Thymidine. Overall lymphocyte proliferation was presented as stimulation indices, which were calculated by dividing the counts per minute (cpm) of antigen-stimulated wells by the cpm of non-stimulated wells.

### Inactivation of E. coli Strains and Antigen Preparation

To obtain inactivated whole cell (WC) antigens from EAEC that was grown as described above, centrifuged and washed twice with 1×PBS. Formaldehyde was added to a concentration of 0.4% and the suspension was incubated at 37°C to inactivate bacteria. After 48 hours of formaldehyde incubation, the inactivated bacteria was centrifuged and washed three times with 1×PBS. To confirm bacterial inactivation, 100 uL of the suspension was plated, incubated for 48 hours at 37°C and analyzed for bacterial growth; no growth was observed. To obtain whole cell sonicated (WCS) antigens, the inactivated bacteria were sonicated 5 times on ice for 20 seconds with 1 minute intervals prior. Protein quantification was performed using the Bradford assay (DC protein assay kit, Bio-Rad Laboratories).

### Statistics

To determine statistical significance in the model, analysis of variance (ANOVA) was performed using the general linear model procedure of Statistical Analysis Software (SAS), and probability value (*P*)<0.05 was considered to be significant. Experiments 1 and 2 were analyzed as (2×2×2) factorial arrangement within a completely randomized design. ANOVA was utilized to determine the main effects of the dietary treatment (nourished vs. malnourished), mouse genotype (wild-type vs. PPAR γ knockout), or the infection status (uninfected vs. infected) and the 2-way and 3-way interactions between dietary treatment, mouse genotype, and infection status. Experiment 3 was analyzed as a completely randomized design. When the model was significant, ANOVA was followed by Fisher’s Protected Least Significant Difference multiple comparison method.

## Results

### The Loss of PPARγ in T Cells Diminishes Growth Retardation during EAEC Infection

**Figure 1:**
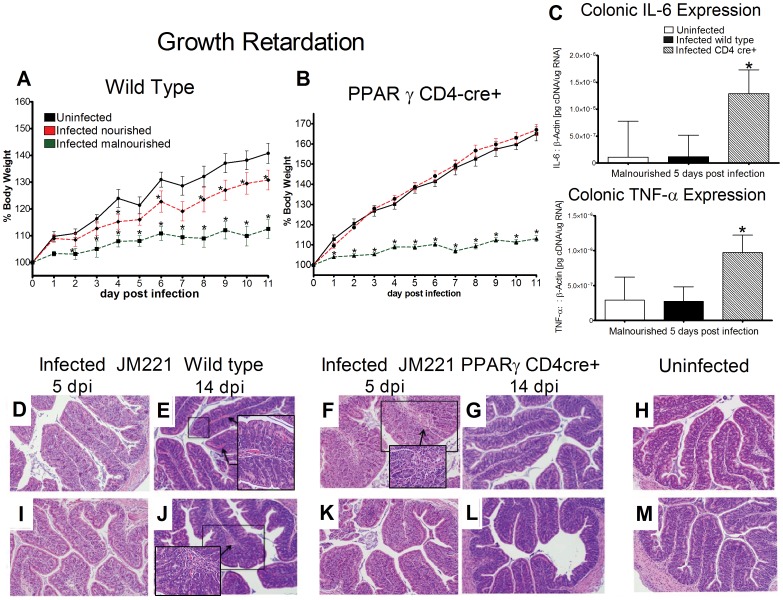
Early beneficial effects of PPARγ deficiency in T cells during enteroaggregative Escherichia coli (EAEC) challenge. Growth retardation in wild type (A) and T cell specific PPARγ deficient mice (B) is expressed as percent growth from day 0 after challenge. Gene expression for IL-6 and TNF-α in colonic tissue of malnourished C57BL/6 and PPARγ CD4cre+ mice was analyzed using quantitative real-time RT-PCR on day 5 PI (C). Representative photomicrographs of colonic specimens of infected mice at 5 or 14 days PI in infected wild type mice (D,E,I,J), infected mice lacking PPARγ expression in T cells (F,G,K,L), and uninfected controls (H,M). The top panel corresponds to nourished mice whereas the bottom panel corresponds to malnourished mice. Original magnification 200×. Boxes and arrows are areas where an amplified image (400×) is provided to emphasize examples of leukocyte infiltration, mucosal thickening, goblet cell hyperplasia, and vasodilation. Mice per group: n = 8. Asterisks indicate values where differences are statistically significant (*p*<0.05).

Detrimental growth shortfalls were observed in infected malnourished mice of all genotypes as early as day three post-infection (PI). Malnourished mice never gained more than 15% of their body weight due to severe protein deficiency. PPARγ null CD4cre+ mice on a control diet grew at rates similar to uninfected mice while nourished infected wild type (WT) mice experienced significant retardation in growth up to 11 days after challenge (Figure 1A–B).

### Histological Analysis Demonstrates Faster Recovery in Mice Lacking PPARγ in T-cells whereas Wild Type Mice Experience Prolonged Inflammation

On day 5 PI CD4cre+ mice had significantly higher levels of mucosal thickening (illustrated by arrow Figure 1F) and leukocyte infiltration (representative of boxed area Figure 1F) while alterations in tissue architecture were negligible in colons of WT mice (Figure S1). Importantly, although significant colonocyte hyperplasia occurred in CD4cre+ mice 5 days PI, the epithelial layer showed no signs of erosion or harmful cell death signifying epithelial barrier integrity was not negatively compromised due to inflammation. Conversely, at dpi 14 WT mice experienced increased levels of mucosal thickness (depicted by arrow Figure 1J), vasodilation (noted by arrows Figure 1E), goblet cell hyperplasia (boxed-in example Figure 1E), and leukocyte infiltration while CD4cre+ mice had no significant signs of inflammation or chronic burden of disease (Figure 1G and L) and resembled uninfected colons (Figure 1 H and M).

### PPARγ Deficiency in T Cells Enhanced Mucosal Effector Response Characterized by Significant Increases in Proinflammatory Gene Expression Early during Infection

At day 5 PI, malnourished CD4cre+ mice expressed significantly elevated levels of IL-6, a pro-inflammatory cytokine responsible for neutrophil and monocyte recruitment early during acute infections [20]. TNF-α, another inflammatory cytokine and activator of neutrophils [21], [22], was also significantly upregulated in colonic tissue from CD4cre+ mice early during infection (Figure 1C).

### Enhanced Antigen-specific Proliferative Recall Responses in Mice Lacking PPARγ in Immune and Epithelial Cells (PPARγ fl/fl Cre+; MMTVcre+) Accompanied by Increased Levels of IL-17 Associated with Dampened EAEC Burden at 14 Days PI

**Figure 2:**
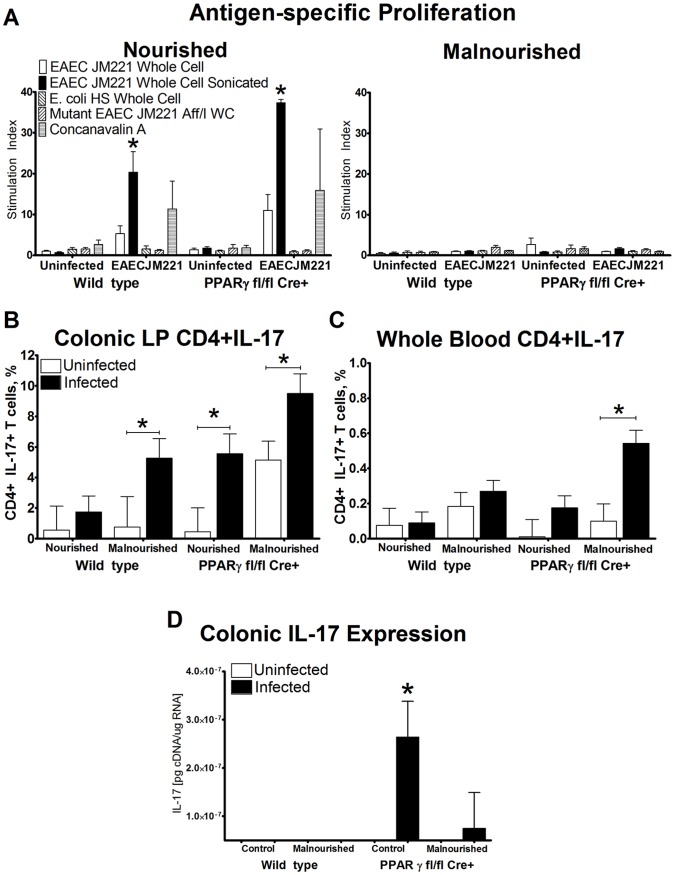
Immune responses during enteroaggregative Escherichia coli (EAEC) infection in peroxisome proliferator-activated receptor γ (PPARγ)-deficient mice associated with bacterial clearance. Antigen specific recall responses of spleenocytes from mice infected with EAEC were measured ex vivo using the lymphocyte blastogenesis test. EAEC JM221 whole cell and whole cell sonicate were used in parallel to two negative controls, *E. coli* HS and mutant EAEC Aff/I strains as well as one positive control, concanavalin A (ConA). Lymphocyte proliferation is expressed stimulation indexes which are calculated by dividing the counts per minute (CPM) of antigen-stimulated wells by the CPM of unstimulated wells (A). IL-17 expression was assessed in colonic lamina propria (B) and whole blood (C) CD4+ T cells by flow cytometry and in the colon by quantitative real time RT-PCR (D) 14 days PI. Mice per group: n = 10. Asterisks indicate values where differences are statistically significant (*p*<0.05) while bars connect groups where comparisons are made.

Overall lymphocyte proliferation was assessed in splenocytes using *ex vivo* antigen stimulation and the incorporation of titrated thymidine in a lymphocyte blastogenesis test. The loss of PPARγ enhanced the magnitude of antigen-specific recall responses to EAEC in nourished mice, whereas malnutrition abrogated responsiveness to antigens or to the mitogen ConA (Figure 2A) regardless of genotype. In addition, PPARγ deficiency led to an increase in colonic IL-17 expression and Th17 responses. IL-17 is one of the first cytokines released during innate responses and plays an essential role in mucosal defense against extracellular bacteria through neutrophil trafficking [23] which is critical for host defense against various pathogens [24]. Tissue from the whole colon was analyzed for IL-17 gene expression 14 days PI. Regardless of infection or diet several mice expressed low basal levels of IL-17 in the colon, however nourished MMTV-cre+ mice expressed significantly elevated levels of colonic IL-17 compared to all other groups (Figure 2D). Malnourished MMTV-cre+ mice also had a higher tendency to express IL-17. Flow cytometric analysis provided evidence that percentages of local CD4+ T cells expressing IL-17 (i.e., Th17 cells) at the colonic mucosa were increased in nourished and malnourished MMTV-cre+ and malnourished WT mice (Figure 2B). The systemic levels of Th17 cells were also significantly elevated in malnourished MMTV-cre+ mice (Figure 2C). These combined results suggest that EAEC infection may induce Th17 responses and the loss of PPARγ enhances the magnitude of Th17 responses. All mice except for the wild-type malnourished completely cleared colonization by day 14 post infection providing evidence that the enhanced effector responses facilitated bacterial clearance (data not shown).

### Pharmacological Blockade of PPARγ Induces Effector and Antimicrobial Mucosal Responses and Facilitates Bacterial Clearance Early during Infection

**Figure 3:**
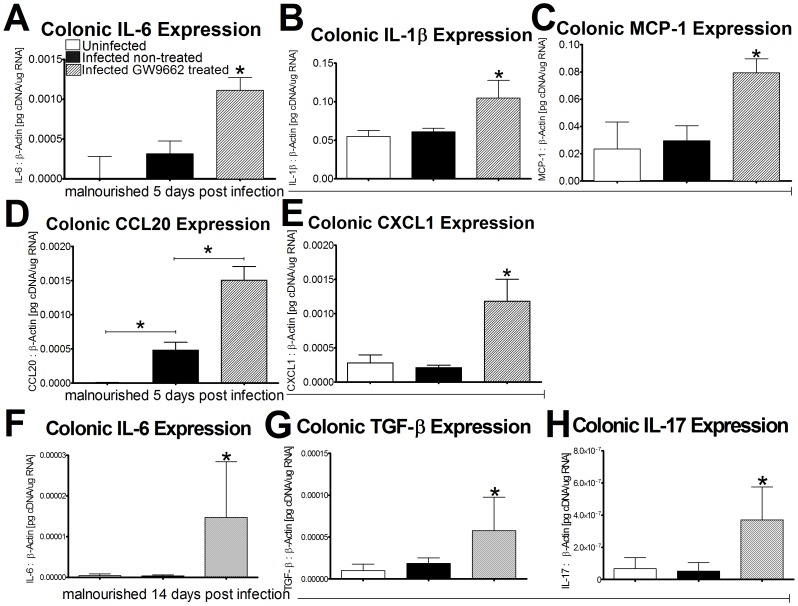
Gene expression suggests a T helper 17 response in mice when peroxisome proliferator-activated receptor γ (PPARγ) is antagonized. Gene expression data from colonic tissue of malnourished C57BL/6 mice was analyzed using quantitative real-time RT-PCR and reported as values normalized to β-actin. IL-6, IL-1β, MCP-1, CCL20, and CXCL1 were quantified at day 5PI (mice per group: n = 10) (A–E) while IL-6, TGF-β, and IL-17 were quantified 14 days PI (n = 10) (F–H). Asterisks indicate values where differences are statistically significant (*p*<0.05) while bars connect groups where comparisons are made.

**Figure 4:**
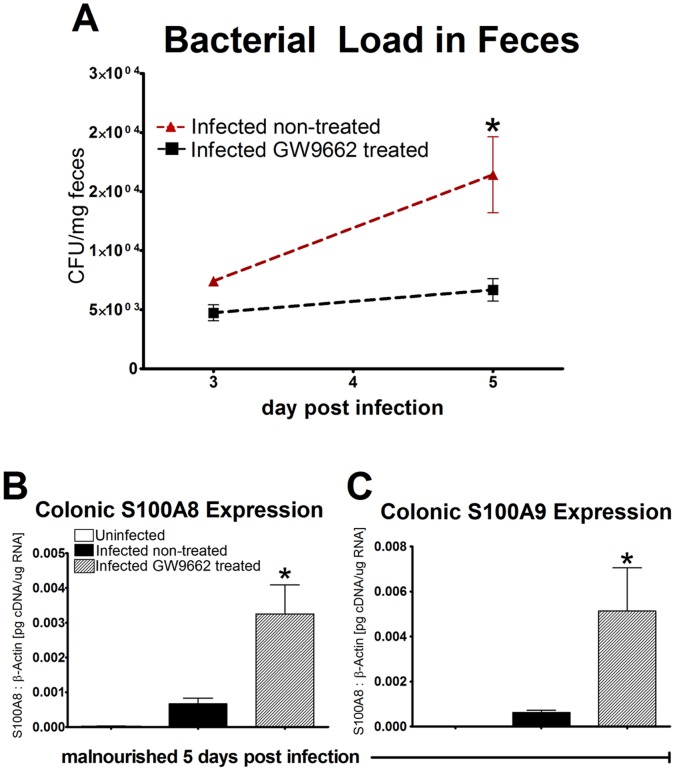
Pharmacological blockade of peroxisome proliferator-activated receptor γ (PPARγ) associated with antimicrobial response and bacterial clearance. Enteroaggregative *Escherichia coli* (EAEC) burden in colon was assessed by quantitative real time RT-PCR using bacterial DNA isolated from feces of infected mice treated with PPARγ antagonist GW9662 (n = 9) or left untreated (n = 9). Data is presented as CFU/mg of tissue. S100A8 and S100A9 gene expression was analyzed in colonic tissue from C57BL/6 malnourished mice at day 5 days PI (n = 10) using quantitative real-time RT-PCR (B and C). S100 proteins are presented as values normalized to β-actin. Asterisks indicate values where differences are statistically significant (*p*<0.05).

Mice that received GW9662 (1 µM) treatment expressed significantly higher levels of proinflammatory cytokines in the colon, including IL-1β, IL-6, CXCL1, and MCP-1, when compared to the untreated group at day 5 PI (Figure 3A–C, E). CCL20 was significantly upregulated in both treated and non-treated infected mice compared to uninfected controls. Additionally, GW9662 treated mice expressed significantly higher levels of colonic CCL20 when compared to the untreated infected mice (*P*<0.0001) (Figure 3D). A significant decrease in IL-10 expression exists in both infected groups at day 5 PI however no significant differences were observed for expression of IL-12p35 and IL-4 (Figure S2 A–C). Proinflammatory cytokine responses in GW9662 treated mice were associated with significantly larger percentages of infiltrating cells to the colonic lamina propria at 5 days post infection. Percentages of CD3+CD4+ T-cells and MHCII+CD11b-CD11c+ DC were significantly higher in GW9662 treated mice while untreated mice experienced higher levels of GR1high+CD11b+ neutrophils. Although no significance in MHCII+F4/80+CD11b+ macrophages was detected between groups, mice treated with GW9662 tended to have higher percentages of this cell phenotype (Figure S3 A–E). Fecal bacterial shedding results demonstrated a significant EAEC burden in infected untreated mice at the peak of infection, 5 days PI, while GW9662 treated mice experienced a mild level of EAEC shedding throughout the duration of infection (Figure 4A). S100A8 and S100A9, proteins that form the antimicrobial peptide complex known as calprotectin, were also significantly upregulated in the colon of mice treated with GW9662 on day 5 PI displaying an enhanced antimicrobial response associated with bacterial clearance (Figure 4B–C). By day 14, calprotectin levels were nearly undetectable in all mice when compared to expression values on day 5 portraying a reduction in antimicrobial responses after the peak of infection (data not shown). Remarkably, by day 14 PI GW9662 treated mice had sustained the stark increase in IL-6 while simultaneously expressing significantly elevated levels of colonic TGF-β and IL-17 further suggesting a Th17 effector response late during infection (Figure 3F–H). Colonic gene expression for IL-10, IL-12p35, and IL-4 revealed no significant differences among groups suggesting that regulatory T cells (Treg), Th1, and Th2 phenotypes were unaffected during infection (Figure S2D–F).

### Neutralizing Anti-IL-17A Antibody Treatment Abrogates the Beneficial Role of GW9662 in Weight Loss and EAEC Burden

**Figure 5:**
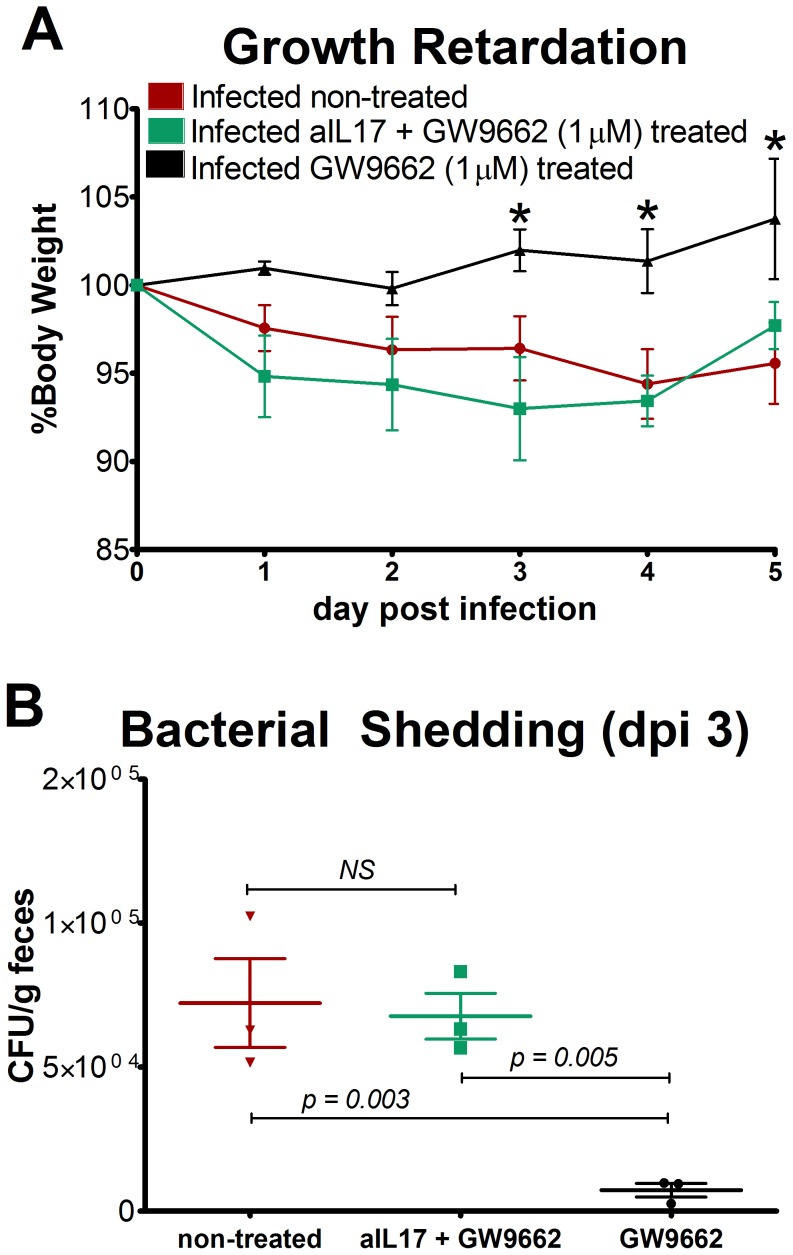
Neutralization of IL-17 abrogates the beneficial effects of GW9662 on weight loss and bacterial burden. Growth retardation in infected wild type mice is expressed as percent growth from day 0 after challenge (A). Enteroaggregative *Escherichia coli* (EAEC) burden in the colon was assessed by quantitative real time RT-PCR using bacterial DNA isolated from feces of infected mice treated with 1 µM PPARγ antagonist GW9662 (n = 3), 50 µg anti-IL17 and 1 µM GW9662 combined (n = 3) or untreated (n = 3). Asterisks indicate values where differences are statistically significant (*p*<0.05), NS signifies no significant difference, and bars are present to indicate significance between groups.

Simultaneous treatment with anti-IL17A and GW9662 (1 µM) resulted in significant differences in body weight beginning on day 3 post-infection (Figure 5A). The pattern of weight loss in mice treated with both anti-IL17A and GW9662 resembled that of untreated mice. Mice solely receiving GW9662 grew at significantly faster rates than the other two groups beginning 3 days after infection. More importantly, significant weight loss coincided with increased bacterial burdens on day 3 PI in mice from non-treated and anti-IL17A+GW9662 groups when compared to GW9662 treatment alone (Figure 5B). These data suggest that the beneficial effects on the host resulting from PPARγ blockade are largely mediated by IL-17A. An additional study performed to assess dose-response effects of GW9662 on IL-17 production revealed that increasing concentrations of GW9662 during infection significantly upregulated colonic IL-17A mRNA expression (Figure S4).

## Discussion

Malnutrition is the most common cause of immunodeficiency worldwide [25]. Protein energy malnutrition is associated with significant impairments in cell-mediated immunity, phagocytosis, cytokine production, and other necessary immune processes that contribute to host protection [26]. Malnourished children are exceptionally susceptible to pathogenic diarrhea that persists and enhances the burden of malnutrition [7], [27]. We provide evidence that malnourished wild type mice exhibited an impaired ability to induce proinflammatory cytokines during EAEC infection. Indeed, cytokine gene expression in the colon of infected, untreated, malnourished, wild type mice was no different than uninfected mice and the lymphoproliferative recall responses of splenocytes to EAEC were impaired. The immunodeficiency observed suggests that these malnourished mice are unable to mount protective innate or adaptive immune responses to EAEC in the gastrointestinal tract. Histopathological analysis indicated minimal inflammation in colons of untreated mice in response to EAEC challenge early during infection even though significantly higher percentages of neutrophils were detected in the colonic lamina propria at that time. By day 14 PI untreated mice began showing signs of chronic pathological burden in the colon noted by elevated bacterial loads, increased goblet cell hyperplasia, and leukocyte infiltration, though the response was still impaired.

PPARγ is a widely expressed transcription factor (i.e., expressed in epithelial cells, macrophages, T and B lymphocytes) and a potent immune modulator that suppresses effector and inflammatory responses [28]. The anti-inﬂammatory effect of PPARγ activation has been extensively studied and known to be mediated by the inhibition of signaling pathways such as NF-κB, AP-1 and STAT [29]. Agonists of PPARγ have shown therapeutic efficacy in mouse models of colitis [19], [30] and clinical inflammatory bowel disease (IBD) [31]. In contrast to IBD, where suppression of inflammation is the desired outcome, our data suggests that a more potent inflammatory and effector response early following infection might be required to clear EAEC infection. We diminished the functionality of PPARγ using a targeted deletion in knockout mice and pharmacological blockade through administration of GW9662, a PPARγ antagonist. Our data indicates that EAEC-infected PPARγ deficient mice developed stronger inflammatory and effector responses towards EAEC early following the challenge ultimately leading to faster recovery from infection. Significant increases in IL-17 were observed locally and systemically while additional potent proinflammatory cytokines (e.g., IL-6, TNF-α, IL-1β) were also significantly upregulated in mice that lacked PPARγ. Epithelial cells secrete a distinct array of proinflammatory mediators including IL-6 [32], TNF-α, and MCP-1 in response to bacterial invasion [33]. During the early stage of infection, PPARγ deficiency significantly upregulated IL-6 and TNF-α expression in malnourished mice. These two proinflammatory cytokines are involved in neutrophil and monocyte recruitment and activation. Likewise, IL-1β, a proinflammatory cytokine responsible for macrophage activation, and CXCL1, a neutrophil-recruiting chemokine, were enhanced significantly [34], [35]. Although IL-1β is translated as an inactive precursor that becomes cleaved by caspase-1 to form its secreted state and caspase-1 activation is dependent upon the formation of a multimolecular scaffold inflammasome, the relative induction of IL-1β mRNA is significant [36]. Additionally, pharmacological blockade of PPARγ increased the colonic expression of MCP-1 five days PI in malnourished mice. MCP-1 expression is induced by IL-1β and remains a primary chemoattractant for monocytes [34]. MCP-1 binds to CCR2 stimulating monocyte differentiation towards the classically activated proinflammatory M1 phenotype [37]. Intestinal epithelial cells also secrete CCL20 in response to enteropathogenic bacteria [38]. EAEC-infected GW9662 treated mice significantly upregulated levels of CCL20 in the colon. CCL20 has potent chemotactic properties contributing to the recruitment of CCR6-expressing cells including dendritic cells, B-cells, and some T cell subsets in mucosal tissue [39], [40]. IL-1β, TNF-α, and IL-17A enhance the release of CCL20 [41]. The interrelationship between these cytokines, whose transcription is regulated by the NF-κB pathway in epithelial and immune cells [42], provides evidence for an orchestrated inflammatory response promoting leukocytic infiltration due to the lack of PPARγ.

This significant increase in pro-inflammatory and effector markers directly correlates to our histological analysis showing significant increases in leukocyte infiltration of T cells, dendritic cells, and macrophages 5 days PI in PPARγ deficient mice. Most significantly, when the bacterial load was quantified over the course of infection, malnourished wild type untreated mice experienced a significant EAEC burden on days 3 and 5 PI while mice administered GW9662 shed low levels of EAEC never amounting values comparable to the WT group. In comparison to the untreated infected counterparts whose innate response was almost undetectable, the significant upregulation of proinflammatory cytokines in malnourished mice lacking PPARγ functionality suggests a beneficial effect of antagonizing PPARγ early during infection to evoke a more potent acute inflammatory response to EAEC. Moreover, PPARγ antagonism was associated with significantly increased levels of calprotectin mRNA expression on day 5 PI. Calprotectin is a phagocyte-derived protein with antimicrobial properties abundantly present in neutrophils, monocytes, and macrophages whose expression is directly correlated with bacteriologically positive infectious diarrhea [43], [44]. Calprotectin is commonly used as a biological marker of neutrophilic intestinal inflammation and infiltrating tissue macrophages, thus the significant upregulation of this protein in GW9662 treated mice provides evidence for an enhanced infiltration of phagocytic leukocytes with antimicrobial properties [45]. The upregulation of calprotectin directly correlates with low levels of EAEC shedding while untreated infected mice that were unable to produce significant amounts of calprotectin experienced enhanced pathological burden at day 5 PI.

In the later phase of infection, WT mice remained immunosuppressed lacking the ability to mount an effective immune response to bacterial invasion noted by the inability to produce proinflammatory genes such as IL-6 and IL-17 at 14 days PI. This too correlated with histopathological findings illustrating a subordinate inflammatory response when compared to the inflammation seen in knockout mice at day 5. Wild type mice continued to lack sufficient responses, however, PPARγ deficient mice transitioned into developing effector responses. By day 14 PI, the significant increase in IL-6 remained and was coupled with upregulation of TGF-β in PPARγ deficient mice. Most importantly, IL-17 was also concurrently expressed in these mice at considerably higher concentrations both locally in the whole colon and colonic lamina propria, as well as systemically in blood by day 14 post-infection. Interestingly, increasing concentrations of GW9662 administered to mice provides evidence for a direct correlation between PPARγ blockade via dose concentration and IL-17A mRNA levels. The colonic gene expression of IL-10, IL-12p35, and IL-4 remained suppressed throughout the duration of infection alluding to the limited impact of colonic Treg, Th1, and Th2 cells in this process [46]–[48]. Recent data is exposing the crucial protective role of IL-17A in immunity to extracellular pathogens because of its abilities to enhance tight junction integrity, mobilize neutrophils, and increase cytokine production by epithelial cells [49]. The primary source of IL-17A varies depending on the immunoregulatory environment. Innate sources of IL-17 appear in a matter of hours after epithelial damage. In the gut, rapid IL-17 production is attributed to γδ T cells, CD8+ T cells, NK cells, NKT cells, and paneth cells [50]–[52], whereas mast cells [53], alveolar macrophages [54] and neutrophils [55] might also produce IL-17 in certain conditions. If infection persists and the initiation of adaptive immunity occurs, the production of IL-17 is more commonly attributed TCRαβ CD4^+^RORγt^+^ Th17 cells [56]. Since the recent discovery of Th17 effector responses, countless studies are concluding the importance of Th17 responses toward extracellular bacteria [57], but to date no studies have examined Th17 responses in EAEC infection. The Th17 population bridges innate and adaptive immunity to produce a robust antimicrobial inflammatory response essential for an effective mucosal and epithelial response to enteric pathogens [58]. Importantly, a Th17 response can effectively recruit and orchestrate neutrophil activation inducing the killing and clearance of extracellular invading pathogens [59]. Our data demonstrated antigen-specific lymphocyte proliferation of splenocytes from infected nourished knockout mice. These results suggest that the blockage of PPARγ was beneficial in generating an effective EAEC-specific effector response from lymphocytes. The data also implicates antigen presentation from innate immune cells was highly effective in mice lacking PPARγ in all immune and epithelial cells. To investigate the effects of enhanced mucosal effector responses on EAEC burden we quantitatively determined the bacterial burden once again at day 14 PI. Malnourished wild type mice endured persistent elevated levels of EAEC while all other mice had ameliorated disease.

A possible explanation for the upregulation of IL-17 in our studies is an early (4–7 day PI) innate IL-17 response initiated by EAEC followed by a robust Th17 response seen 10–14 days PI similar to previously characterized responses to other pathogenic bacteria [60]. Importantly, CCR6, whose ligand is CCL20, has been established as the homing receptor for Th17 cells and regulates the migration of Th17 cells in the intestine [61]. The significant increase in colonic CCL20 expression in mice treated with GW9662 during infection combined with flow data displaying significantly higher percentages of DC and T cells in the colonic lamina propria further supports an enhanced active recruitment of these cells early during infection. In response to bacterial infection, dendritic cells produce IL-6, TGF-β, and IL-1β that drive IL-17 production from innate lymphocytes [62]. A limitation of our colonic IL-17 expression analyses is that we have not characterized the cell that produces this cytokine. However, our flow cytometry data indicate systemic and mucosal production of IL-17 by CD4+ T cells, suggesting for the first time a role for Th17 responses in the clearance of EAEC infections. We observed significantly higher levels of CD4+ T cells present in the colonic lamina propria of mice treated with GW9662 illustrating an early recruitment of T cells. Th17 differentiation is induced by a combination of TGF-β and IL-6 or IL-21 along with the expression of the RORγt transcription factor [63]. Colonic gene expression data from day 14 PI showed significant upregulation of IL-6 and TGF-β providing further evidence that Th17 cells are present late during EAEC infection. Treatment of mice with GW9662 and anti-IL-17 abrogated the beneficial effects of GW9662 on weight loss and EAEC burden, suggesting that the blockade of PPAR γ ameliorates EAEC infection and disease through an IL-17-dependent mechanism. Future studies will characterize the origin of mucosal IL-17 to determine whether this is an EAEC-mediated Th17 response or an innate response as well as the potential role of the CCL20/CCR6 pathway in mediating infiltration of Th17 cells in the colonic LP.

Previous studies suggest potential harmful effects of inflammation during EAEC infection in healthy adult volunteers due to transmigration of neutrophils disrupting the epithelial barrier [13], [64]. In contrast, we show that malnourished mice were unable to generate effective innate or adaptive responses towards EAEC on their own which resulted in higher bacterial burden throughout the course of infection. We speculate that mucosal effector responses in the malnourished mouse were impaired due to malnutrition and were therefore unable to control EAEC. By blocking PPARγ, we promoted proinflammatory cytokine production and leukocyte infiltration at day 5 PI. Pharmacological blockade of PPARγ through administration of increasing concentrations of GW9662 provides evidence for a direct correlation between dose concentration and IL-17A mRNA levels. More importantly, PPARγ deficient mice were able to generate a Th17 effector response by day 14 post-infection. These responses were likely involved in bacterial clearance.

In conclusion, we report for the first time the importance of Th17 responses in clearing EAEC infections and the beneficial role for PPARγ blockade and subsequent upregulation of colonic effector and inflammatory responses during EAEC infection. More specifically, PPARγ blockade significantly enhanced lymphoproliferative recall responses, upregulated expression of IL-17, anti-microbial peptides and inflammatory cytokines at the colonic mucosa, and decreased EAEC fecal shedding. Thus, PPARγ antagonism represents a novel host-targeted therapeutic approach for EAEC infections.

## Supporting Information

Figure S1 **Histological analysis of colonic tissue provides evidence for higher effector response in mice lacking peroxisome proliferator-activated receptor (PPAR) γ early in infection.** Cross sections from wild-type (WT) and T cell-specific PPAR γ null (CD4cre+) mouse colonic tissue were analyzed for leukocyte infiltration and mucosal thickening on days 5 and 14 post infection (DPI). Each mouse was scored based on a numeric system from 0 to 4; 0 is representative of an uninfected mouse and 4 is indicative of severe changes in the mucosal architecture. Asterisks indicate values where differences are statistically significant compared to uninfected control scores (*p*<0.05).
(TIF)

Figure S2 **Colonic gene expression data suggests T helper (Th)1, Th2, and regulatory T (Treg) cell phenotypes are not dramatically impacted during EAEC infection.** Gene expression levels for cytokines IL-10, IL-12, and IL-4 were quantified in colonic tissue from C57BL/6 malnourished mice at day 5 (A–C) and 14 (D–F) post-infection (mice per group: n = 10) using quantitative real-time RT-PCR. Data is presented as values normalized by β-actin. Asterisks indicate values where differences are statistically significant (*p*<0.05).
(TIF)

Figure S3 **Flow cytometry analysis of leukocytic infiltration during EAEC infection suggests different cell phenotypes when PPARγ functionality is diminished.** Colonic lamina propria lymphocytes isolated from infected mice treated with 1 µM GW9662 (n = 3) or left untreated (n = 3) were stained with fluorochrome-conjugated primary antibodies and analyzed using FACS Diva software. Data are presented as percentages as viable cells, CD45+, or CD45+MHCII+ (indicated in the y-axis). Asterisks indicate values where differences are statistically significant (*p*<0.05).
(TIF)

Figure S4 **Increasing doses of GW9662 induce enhanced levels of IL-17 mRNA expression.** Gene expression data from colonic tissue of malnourished C57BL6 mice that received different doses of GW9662 was analyzed on day 5 post infection using quantitative real-time RT-PCR. Data are reported as values normalized to β-actin (mice per group: n = 3). Asterisks indicate values where differences are statistically significant (*p*<0.05) while bars indicate groups where comparisons are made.
(TIF)

Table S1 **Composition table of purified experimental diets.**
(DOCX)

Table S2 **Nucleotide sequences, base pair length, and accession number used to design primers for quantitative real-time RT-PCR.**
(DOCX)
