# Supplementary figures and images for: The Role of Peroxisome Proliferator-Activated Receptor γ in Immune Responses to Enteroaggregative Escherichia coli Infection
Source: PLoS One. 2013 Feb 28;8(2):e57812. doi: 10.1371/journal.pone.0057812 (PMC3585146; doi:10.1371/journal.pone.0057812)

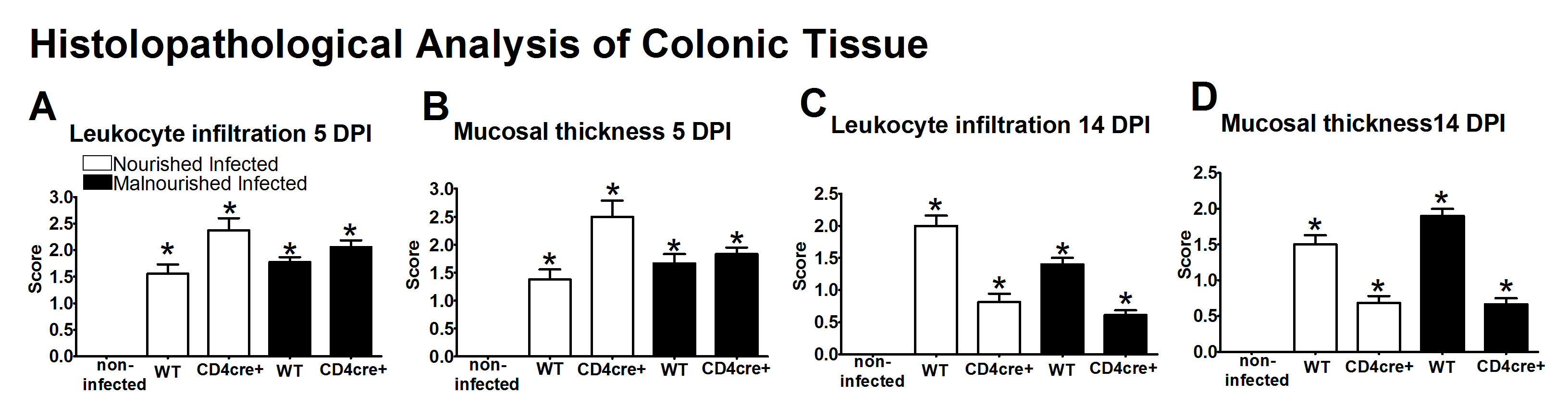

Supplement: Figure S1 — Histological analysis of colonic tissue provides evidence for higher effector response in mice lacking peroxisome proliferator-activated receptor (PPAR) γ early in infection. Cross sections from wild-type (WT) and T cell-specific PPAR γ null (CD4cre+) mouse colonic tissue were analyzed for leukocyte infiltration and mucosal thickening on days 5 and 14 post infection (DPI). Each mouse was scored based on a numeric system from 0 to 4; 0 is representative of an uninfected mouse and 4 is indicative of severe changes in the mucosal architecture. Asterisks indicate values where differences are statistically significant compared to uninfected control scores (p<0.05). (TIF) [file pone.0057812.s001.tif]

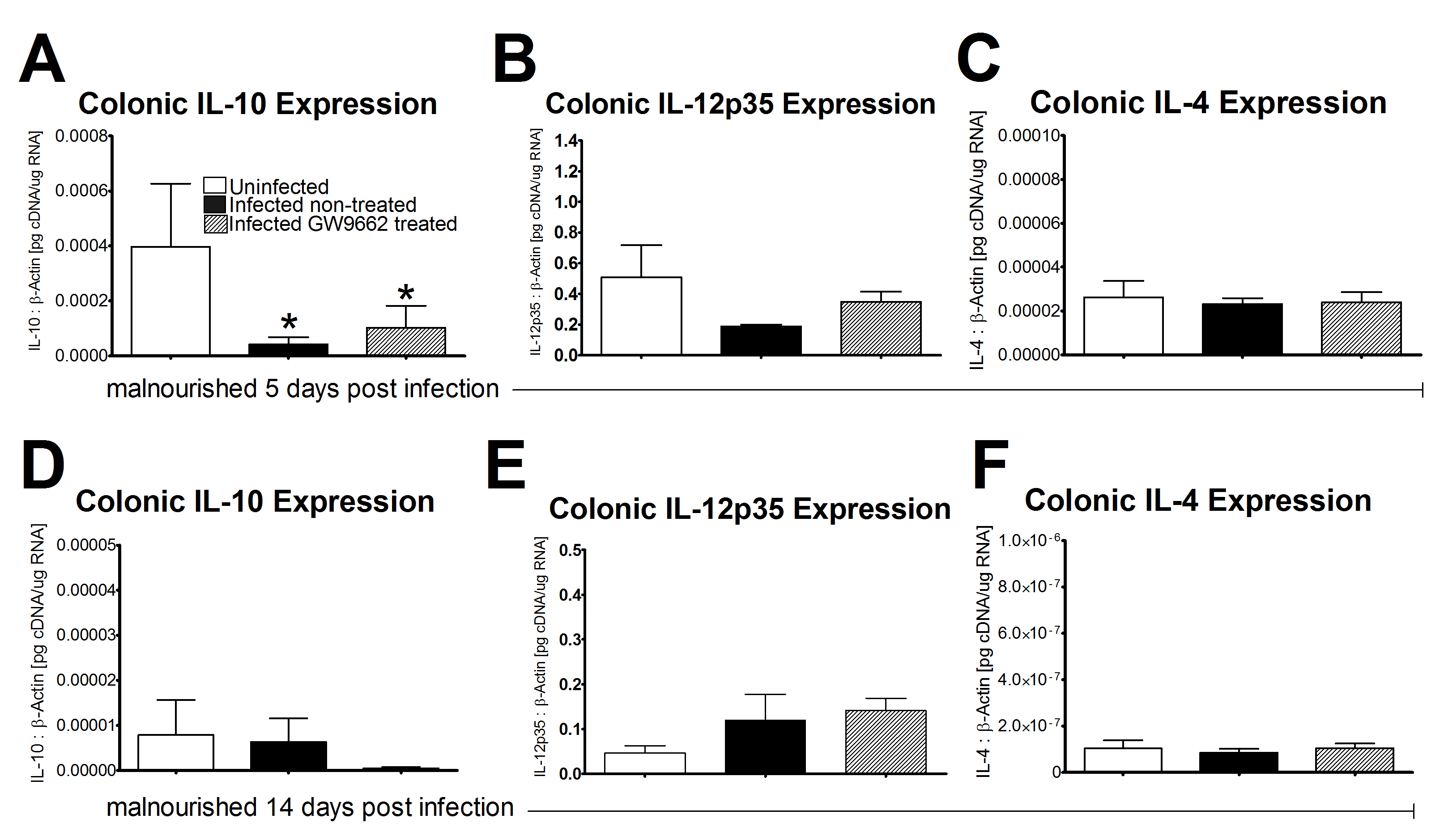

Supplement: Figure S2 — Colonic gene expression data suggests T helper (Th)1, Th2, and regulatory T (Treg) cell phenotypes are not dramatically impacted during EAEC infection. Gene expression levels for cytokines IL-10, IL-12, and IL-4 were quantified in colonic tissue from C57BL/6 malnourished mice at day 5 (A–C) and 14 (D–F) post-infection (mice per group: n = 10) using quantitative real-time RT-PCR. Data is presented as values normalized by β-actin. Asterisks indicate values where differences are statistically significant (p<0.05). (TIF) [file pone.0057812.s002.tif]

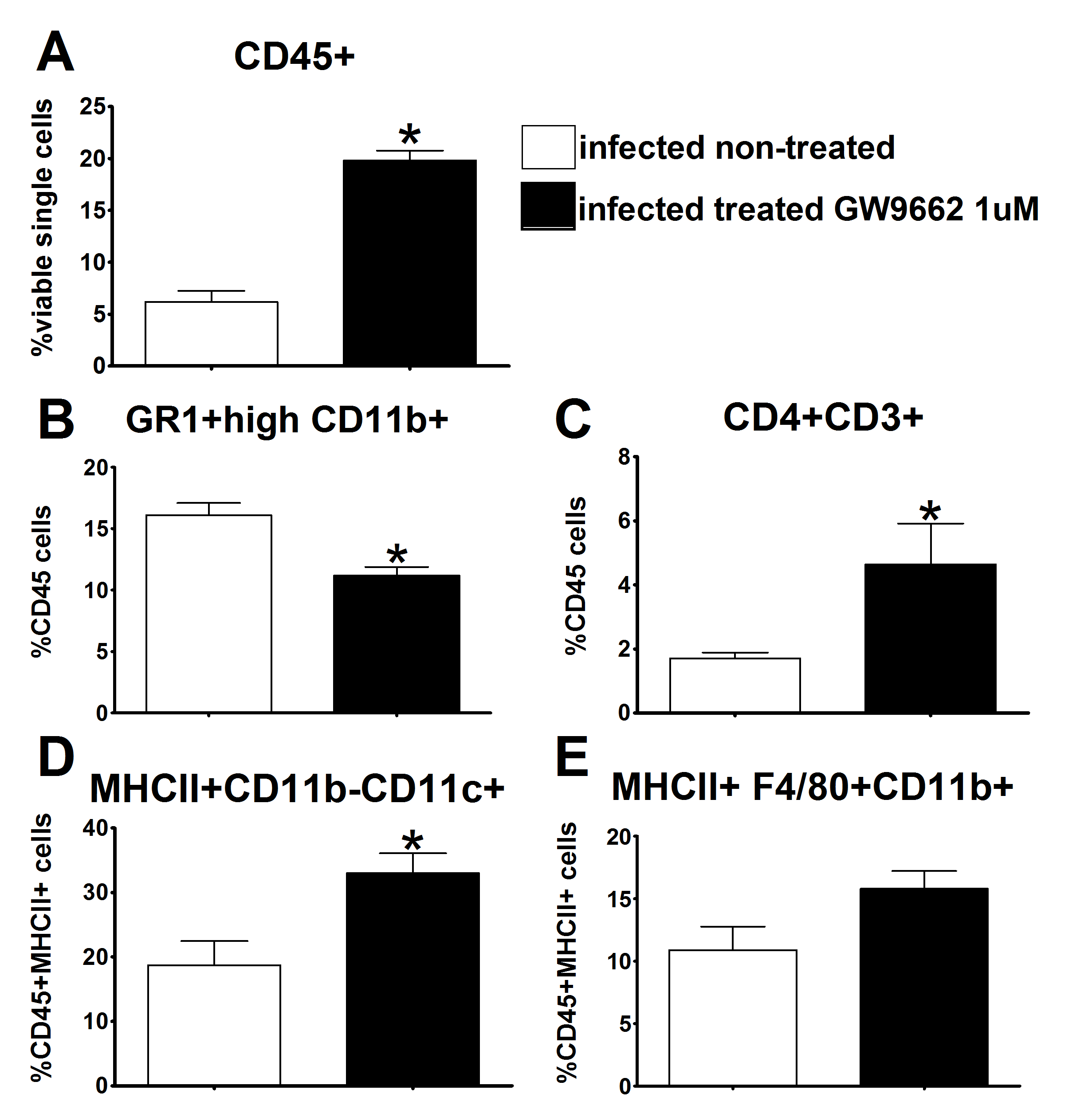

Supplement: Figure S3 — Flow cytometry analysis of leukocytic infiltration during EAEC infection suggests different cell phenotypes when PPARγ functionality is diminished. Colonic lamina propria lymphocytes isolated from infected mice treated with 1 µM GW9662 (n = 3) or left untreated (n = 3) were stained with fluorochrome-conjugated primary antibodies and analyzed using FACS Diva software. Data are presented as percentages as viable cells, CD45+, or CD45+MHCII+ (indicated in the y-axis). Asterisks indicate values where differences are statistically significant (p<0.05). (TIF) [file pone.0057812.s003.tif]

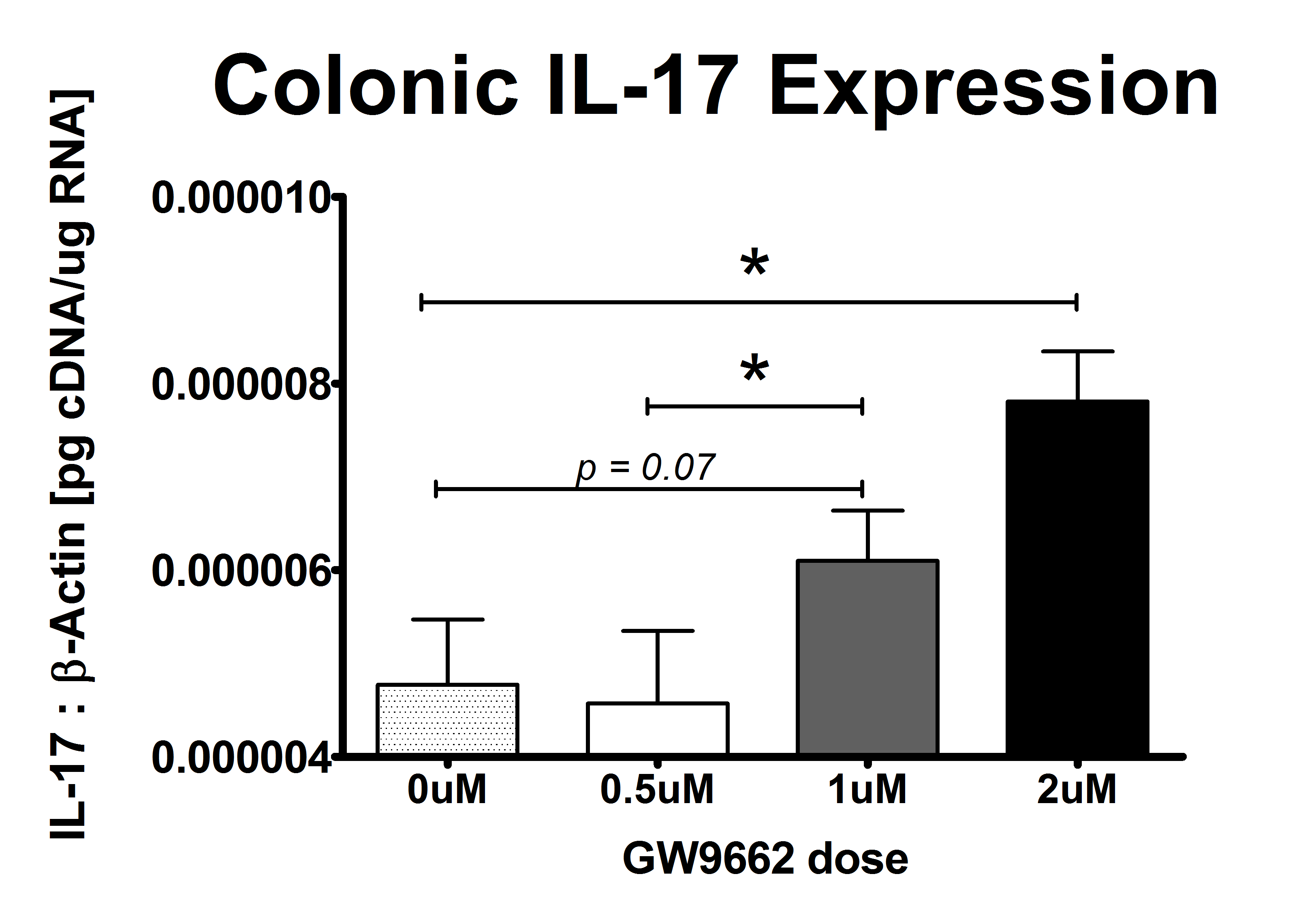

Supplement: Figure S4 — Increasing doses of GW9662 induce enhanced levels of IL-17 mRNA expression. Gene expression data from colonic tissue of malnourished C57BL6 mice that received different doses of GW9662 was analyzed on day 5 post infection using quantitative real-time RT-PCR. Data are reported as values normalized to β-actin (mice per group: n = 3). Asterisks indicate values where differences are statistically significant (p<0.05) while bars indicate groups where comparisons are made. (TIF) [file pone.0057812.s004.tif]
