# Supplementary material for: The Role of Peroxisome Proliferator-Activated Receptor γ in Immune Responses to Enteroaggregative Escherichia coli Infection
Source: PLoS One. 2013 Feb 28;8(2):e57812. doi: 10.1371/journal.pone.0057812 (PMC3585146; doi:10.1371/journal.pone.0057812)
Supplement: Table S1 — Composition table of purified experimental diets. (DOCX) [file pone.0057812.s005.docx]

***Table S1.*** ***Composition table of purified experimental diets.***

|  | AIN-93G Control | 2% Protein Diet |
| --- | --- | --- |
| Ingredient | g/Kg | |
| Casein | 200 | 23 |
| L-Cystine | 3 | 0.35 |
| Corn Starch | 397.486 | 582.746 |
| Maltodextrin | 132 | 132 |
| Sucrose | 100 | 100 |
| Soybean Oil | 70 | 70 |
| Cellulose | 50 | 50 |
| Mineral Mix (AIN-93G-MX)^a^ | 35 | 13.39 |
| Mineral Mix, w/o Ca & P | --- | 12.5 |
| Calcium Carbonate | --- | 3.4 |
| Vitamin Mix (AIN-93-VX)^b^ | 10 | 10 |
| Choline Bitartrate | 2.5 | 2.5 |
| TBHQ antioxidant | 0.014 | 0.014 |
| Food Color | --- | 0.1 |

a. Supplied per kg of diet: 3 g nicotinic acid, 1.6 g calcium pantotenate, 0.7 g pyridoxine HCl, 0.6 g thiamin HCl, 0.6 g riboflavin, 0.2 g folic acid, 0.02 g D-biotin, 2.5 g vitamin B_12_ (0.1% in mannitol), 15 g DL-α tocopheryl acetate (500 IU/g), 0.8 g vitamin A palmitate (500,000 IU/g), 0.2 g vitamin D_3_(cholecalciferol, 500,000 IU/g), 0.075 g vitamin K (phylloquinone), and 974.705 g sucrose.

b. Supplied per kg of diet: 357 g calcium carbonate, 196 g potassium phosphate monobasic, 70.78 g potassium citrate, 74 g sodium chloride, 46.6 g potassium sulfate, 24.3 g magnesium oxide, 6.06 g ferric citrate, 1.65 g zinc carbonate, 0.63 g manganous carbonate, 0.31 g cupric carbonate, 0.01 g potassium iodate, 0.01025 g sodium selenate, 0.00795 g ammonium paramolybdate, 1.45 g sodium meta-silicate, 0.275 g chromium potassium sulfate, 0.0174 g lithium chloride, 0.0815 g boric acid, 0.0635 g sodium fluoride, 0.0318 g nickel carbonate, hydroxide, tetrahydrate, 0.0066 g ammonium vanadate, and 220.716 g sucrose.
