# Supplementary material for: The Role of Peroxisome Proliferator-Activated Receptor γ in Immune Responses to Enteroaggregative Escherichia coli Infection
Source: PLoS One. 2013 Feb 28;8(2):e57812. doi: 10.1371/journal.pone.0057812 (PMC3585146; doi:10.1371/journal.pone.0057812)
Supplement: Table S2 — Nucleotide sequences, base pair length, and accession number used to design primers for quantitative real-time RT-PCR. (DOCX) [file pone.0057812.s006.docx]

| ***Table S2.*** ***Nucleotide sequences, base pair length, and accession number used to design primers for quantitative real-time RT-PCR.*** | | | |
| --- | --- | --- | --- |
| Primer | Sequence | Length | Accession number |
| mβ-actin F | 5’-CCCAGGCATTGCTGACAGG-3’ | 141 | X03672 |
| mβ-actin R | 5’-TGGAAGGTGGACAGTGAGGC-3’ |  |  |
| mIL-1β F | 5'-TATCACTCATTGTGGCTG-3' | 76 | NM_008361.3 |
| mIL-1β R | 5'-ATGTCCTCATCCTGGAAG-3' |  |  |
| mIL-17a F | 5'-CCAGACGGCCCTCAGATTAC-3' | 103 | NM_010552.3 |
| mIL-17a R | 5'-CACTTGGCCTCCCAGATCAC-3' |  |  |
| mIL-6 F | 5'-TTTCCTCTGGTCTTCTGGAG-3' | 112 | NM_031168.1 |
| mIL-6 R | 5'-CTGAAGGACTCTGGCTTTGT-3' |  |  |
| mMCP-1 F | 5'-CTGCCTAATCCACAGACTG-3' | 142 | AJ238892 |
| mMCP-1 R | 5'-GCCTGAACAGCACCACTA-3' |  |  |
| mTNF-α F | 5’-CGTGCTCCTCACCCACAC-3’ | 133 | M13049 |
| mTNF-α R | 5’-GGGTTCATACCAGGGTTTGA-3’ |  |  |
| mTGF-β F | 5′CAACTTCTGTCTGGGACCCT3 | 150 | NM_011577 |
| mTGF-β R | 5′TAGTAGACGATGGGCAGTGG3′ |  |  |
| aap F | CTT GGG TAT CAG CCT GAA TG | 310 | FN554767.1 |
| aap R | AAC CCA TTC GGT TAG AGC AC |  |  |
| mIL-12p35 F | CAGATAGGAAACAAAGAAAGAT | 129 | NM_001159424 |
| mIL-12p35 R | GGAGATGAGATGTGATGG |  |  |
| mIL-4 F | GCTATTGATGGGTCTCAACC | 112 | NM_021283.1 |
| mIL-4 R | GCCGATGATCTCTCTCAAGT |  |  |
| mIL-10 F | GGGCTTCTTTCTAAATAGTTC | 135 | NM_010548.1 |
| mIL-10 R | GATCTTAGCTAACGGAAACA |  |  |
| CCL20 F | CAGCACTGAGCACATCTACT | 128 | NM_016960.2 |
| CCL20 R | CAGCAGCACCGACATCAA |  |  |
| S100A8 F | CGACACCTTCCATCAATACTCT | 92 | NM_009114 |
| S100A8 R | TTGCCAACTGTGCTTCCA |  |  |
| S100A9 F | GACATCAATAGTGACAAT | 143 | NM_013650 |
| S100A9 R | TATTCTGTAGACATATCCA |  |  |
| CXCL1 F | GTCATAGCCACACTCAAG | 93 | NM_008176 |
| CLCL1 R | GACACCTTTTAGCATCTTTTG |  |  |
